# How the pan-genome is changing crop genomics and improvement

**DOI:** 10.1186/s13059-020-02224-8

**Published:** 2021-01-04

**Authors:** Rafael Della Coletta, Yinjie Qiu, Shujun Ou, Matthew B. Hufford, Candice N. Hirsch

**Affiliations:** 1 Department of Agronomy and Plant Genetics, University of Minnesota, St. Paul, MN 55108 USA; 2 Department of Ecology, Evolution, and Organismal Biology, Iowa State University, Ames, IA 50011 USA

## Abstract

Crop genomics has seen dramatic advances in recent years due to improvements in sequencing technology, assembly methods, and computational resources. These advances have led to the development of new tools to facilitate crop improvement. The study of structural variation within species and the characterization of the pan-genome has revealed extensive genome content variation among individuals within a species that is paradigm shifting to crop genomics and improvement. Here, we review advances in crop genomics and how utilization of these tools is shifting in light of pan-genomes that are becoming available for many crop species.

**Supplementary Information:**

The online version contains supplementary material available at 10.1186/s13059-020-02224-8.

## Introduction

Crop improvement is needed now more than ever with challenges associated with feeding an ever-expanding population under increasingly variable growth conditions. The ability to produce crops that meet societal needs is enhanced by a thorough understanding of the genome of a species. Genomic resources expand the toolbox available for plant breeding and crop improvement efforts. Various tools have risen in popularity for plant breeding, in some cases as short-lived bandwagons and others as paradigm shifts in crop improvement [1, 2]. Within crop genomics, advances relevant to crop improvement have primarily been in marker (e.g., Illumina single nucleotide polymorphism (SNP) chips, kompetitive allele-specific PCR (KASP) assays, genotyping-by-sequencing (GBS)) and sequencing (e.g., Illumina, PacBio, Nanopore) technology. Recent innovations are driving a paradigm shift in which the extent and relevance of structural variation within the pan-genome of crop species are now being considered.

**Fig. 1:**
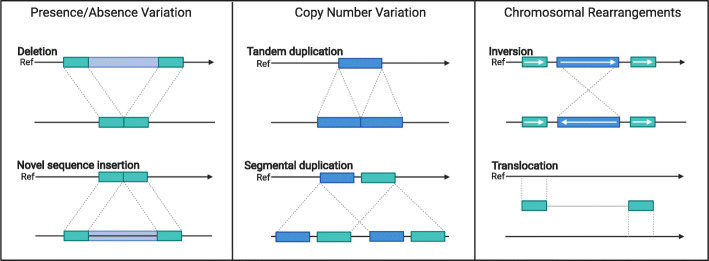
Diagrams of structural variants that can be found in crop genomes

Access to plant genome assemblies in the early 2000s revolutionized thinking about the biology of crops and plant breeding [3–5]. These early assemblies allowed for a deeper understanding of the diversity in plant species, primarily at the level of SNPs [6–9]. However, after a short while, it became obvious that single-reference assemblies represent only a small fraction of species-wide genomic space [10–13]. Extensive structural variants (SVs) (e.g., presence-absence variation (PAV), copy number variation (CNV), and chromosomal rearrangements; Fig. 1) were discovered, with the first two classes contributing to the variation in genome content. Within species, genomes vary in both gene content (e.g., tandem duplicated genes, CNVs dispersed throughout the genome, and PAVs of genes) and repetitive portions of the genome (e.g., transposable elements, knob repeats, centromere repeats). In characterizing this variation, the genomic fraction common to all individuals within a species has been termed the “core” genome and the variable fraction the “dispensable” genome.

There are many mechanisms that can generate a structural variation. For example, transposable elements (TEs) can replicate themselves in a genome and can also capture and carry gene sequences to new genomic locations [14–16]. This process can cause significant disruption of the coding portion of the genome [15, 16]. Additionally, structural variation can be introduced through errors during meiotic recombination [17], such as non-allelic homologous recombination (unequal recombination [18]) and double-strand break repair via single-strand annealing [19]. Finally, PAVs can be created, especially in plants, through differential genome fractionation across genotypes following a whole-genome duplication event [20], although in maize, a paleopolyploid, it was shown that this phenomenon played a limited role in creating SVs among elite temperate germplasm [21].

The generation of multiple, reference-quality genome assemblies per crop species is now a reality [22–24]. Our way of thinking about crop genomics is changing as we gain a deeper understanding of the structural variation within the pan-genome. Initial efforts to dissect the genetic architecture of traits (e.g., quantitative trait locus (QTL) mapping and genome-wide association studies (GWAS)) and genomic prediction efforts have relied primarily on SNP markers. The structural variation that has been uncovered in the pan-genome era necessitates a reevaluation of the determinants of phenotype. To date, structural variation has already been associated with environmental adaptation such as tolerance of abiotic and biotic stress [25–28] and flowering time ([29, 30]; for an extensive review, see [31]). Additionally, plant domestication traits such as non-shattering [32] and changes in plant architecture [33, 34] are caused by SVs. For example, a TE insertion ~ 60 kb upstream of the maize *tb1* gene played an important role in changing maize architecture during its domestication [35]. In fact, SVs in non-coding regions have been shown in many instances to influence gene expression of nearby genes [36, 37]. Given the breadth of traits affected by SV, their characterization is important for crop domestication and improvement and will facilitate future efforts in these areas.

Crop genomics has transitioned from the era of a single reference genome to a time when we now have access to tens or hundreds of reference-quality genome assemblies within a species (Table 1). This article reviews previous crop genomic efforts relevant to crop improvement and expected advances in light of recent progress in characterizing structural variation at the pan-genome level.

**Table 1:** Summary of plant species with pan-genomes currently available

Species	Estimated mean DNA amount (C-value)^a^	Method for pan-genome construction	Number of accessions	Sequencing method	Reference
*Brachypodium distachyon*	*B. distachyon*, 0.32 pg *B. stacei*, 0.28 pg *B. hybridum*, 0.63 pg	De novo assembly^b^	54	Illumina HiSeq	[38]
*B. distachyon*, *Brachypodium hybridum*, *Brachypodium stacei*		De novo assembly^b^	57	Illumina HiSeq PacBio	[39]
*Medicago truncatula*	0.47 pg	De novo assembly^b^	15	Illumina HiSeq	[40]
*Oryza sativa* (Asian rice)	*O. rufipogon*, 0.46 pg *O. nivara*, 0.47 pg *O. barthii*, 0.60 pg *O. glaberrima*, 0.53 pg *O. sativa*, 0.50 pg	Iterative mapping and assembly^c^	1483	Illumina HiSeq	[41]
*O. sativa* (Asian rice)		Map to pan^d^	3010	Illumina HiSeq PacBio	[42]
*O. sativa*/*Oryza rufipogon* (Asian and common wild rice)		De novo assembly^b^	66	Illumina HiSeq	[43]
*O. sativa* (Asian rice)		De novo assembly^b^	12	PacBio	[44]
*O. rufipogon*/*O. nivara*/*O. barthii*/*O. glaberrima* (wild rice and African rice)		De novo assembly^b^	4	PacBio	[45]
*Juglans* ssp. (walnuts)	0.64 pg	De novo assembly^b^	6	Illumina HiSeq	[46]
*Malus domestica*/*M. sieversii*/*M. sylvestris* (apple and wild apple progenitors)	*M. domestica*, 0.88 pg *M. sieversii*, 0.75 pg *M. sylvestris*, 0.78 pg	Iterative mapping and assembly^c^	91	Illumina HiSeq PacBio	[47]
*Brassica oleracea*, *Brassica macrocarpa* (cultivated and wild cabbage)	*B. oleracea*, 0.9 pg *B. macrocarpa*, not available *B. napus*, 1.10 pg	Iterative mapping and assembly^c^	10	Illumina HiSeq	[48]
*Brassica napus* (oilseed)		Map to pan^d^	9	Illumina Hiseq PacBio	[22]
*Solanum lycopersicum* (tomato)	1.06 pg	Map to pan^d^	725	Illumina NextSeq	[49]
		De novo assembly^b^	100 (14 assembled)	Illumina NextSeq Nanopore	[23]
*Glycine soja* (wild soybean)	*G. soja*, 1.10 pg *G. max*, 1.13 pg	De novo assembly^b^	7	Illumina HiSeq	[12]
*Glycine max* (soybean)		De novo assembly^b^	29	Illumina HiSeq PacBio	[24]
*Zea mays* (maize)	2.7 pg	Novel transcript assembly^e^	503	Illumina HiSeq	[10]
		De novo assembly^b^	6	Illumina HiSeq	[50]
*Capsicum annuum* (pepper)	3.16 pg	Iterative mapping and assembly^c^	383	Illumina HiSeq	[51]
*Helianthus annuus* (sunflower)	3.67 pg	Map to pan^d^	493	Illumina HiSeq	[52]
*Triticum aestivum* (bread wheat)	24.65 pg	Iterative mapping and assembly^c^	19	Illumina HiSeq	[53]

^a^The mean 1C (pg) value was obtained from the plant DNA C-values Database (https://cvalues.science.kew.org), 1 pg = 978 Mb [54]

^b^Assemble and annotate each genome separately and identify the variable regions

^c^De novo assemble individual genome and then compare the assembled genome to the reference genome to capture the gene information that is not present in the reference genome

^d^Map reads to the reference genome, perform de novo assembly using unmapped reads, and incorporate the information into the reference genome

^e^De novo assembly of short reads to capture transcript diversity

### Assembly and bioinformatic advances allow characterization of crop pan-genomes

#### Advances in crop genome assembly technology

Over the last two decades, advances in sequencing technology and assembly algorithms have profoundly affected our understanding of the complexity and structure of genomes. Crops were among the first species with assembled genomes given their economic importance and the relevance of genomic information to breeding. The earliest model crop genomes were assembled with Sanger sequencing, BAC-by-BAC approaches, and overlap-layout-consensus (OLC) assemblers (e.g., rice [3], maize [4], sorghum [55], soybean [56], and grape [57]). Subsequent crop reference genomes increasingly relied on next-generation sequencing (e.g., potato [58]) with some assembled entirely from paired-end and mate-pair Illumina data and de Bruijn graph approaches (e.g., barley, wheat [59, 60]). These crop reference assemblies were, in many cases, rapidly followed by large resequencing studies in which short-read data were generated for additional individuals and mapped to the reference to characterize species-level diversity (e.g., rice [42, 61, 62], maize [6], soybean [63]).

Within the last 5 years, the reduced cost of Illumina sequencing and improved assembly algorithms facilitated de novo assembly of multiple accessions per crop using low-cost short-read data (e.g., maize-PH207 [64], maize-W22 [65], maize-HZS [66], maize-Flint genomes [50], rice genomes [43, 67], soybean genomes [12]). While this approach has generated highly complete and contiguous assemblies of low-copy genic regions, the more repetitive, TE-rich regions of the genome have proven recalcitrant to assembly with short reads, resulting in numerous gaps and partial assembly in these regions.

Recently, the maturation of long-read technology has facilitated much more contiguous and complete assembly of crop genomes [68–72] and, in some cases, multiple long-read-based assemblies within a single species [23, 24]. These assemblies are already facilitating discoveries of the relevance of non-coding and regulatory variation to agronomic traits, among other important discoveries [73, 74]. Sequence data continues to improve rapidly with sequence output increasing steadily and error rates decreasing (e.g., PacBio HiFi libraries), thereby diminishing the cost of assembly and increasing the utility of long-read assemblies for uncovering agronomically relevant variation across lines within crop species.

#### Characterizing structural variation based on a single reference genome

Methods to detect structural variation began to appear shortly after the publication of the first genome assemblies and have continued to develop as sequencing technologies have advanced (for comprehensive reviews, see [75, 76]). Early efforts to characterize CNV/PAV across species relied on hybridization arrays (e.g., comparative genomic hybridization (CGH)) that were based on probes often designed using only sequence from an initial reference genome assembly [11, 13, 19]. While array-based approaches are relatively inexpensive and high-throughput, they do have limitations. For example, once an array is developed, it is a static instrument, and newly identified loci of interest are not characterized. Additionally, when probes are based on a single reference sequence, ascertainment bias can be observed (i.e., hybridization efficiency diminishes when samples are more divergent from the reference individual).

As short-read resequencing decreased in cost and became commonplace, whole-genome sequencing (WGS) approaches were more frequently used to characterize CNV/PAV in crops [77–79]. These approaches for detecting CNV/PAV fall into three main categories: read depth, read pair, and split read [80]. With read-depth methods, short reads are mapped to a reference, and the relative depth of sequence at a locus serves as a proxy for copy number in a given individual [80]. Read-pair methods identify CNV/PAV based on discrepancies in the distance between paired-end sequences relative to their distance in the reference assembly [80]. Split-read methods detect SVs that interrupt the sequence within short reads [80].

The use of whole-genome sequencing allowed for characterization of a greater breadth of variants than hybridization arrays, but this approach suffers similar limitations: (1) sequence from loci that are missing in the reference genome due to either incomplete assembly or true biological absence does not map and remains uncharacterized, (2) divergent reads map less efficiently, and (3) uneven coverage bias of short-read sequencing can result in inaccuracies [81]. These shortcomings have been addressed to some extent through the assembly of unmapped reads [10, 48] and through the use of pseudo-references in which line-specific SNPs are introduced into the reference to increase mapping efficiency [82]. Characterization of structural variation in the repetitive fraction of the genome is particularly challenging with short-read resequencing data because mapping and assembly of unmapped reads are particularly inefficient and unreliable in these regions.

New approaches have rapidly developed for CNV/PAV characterization that leverage recently developed library preparation techniques and the maturation of single-molecule, long-read sequencing (comprehensively reviewed in [75]). For example, connected-molecule approaches (10x, Hi-C, Strand-Seq) can characterize long-range information using short reads through the development of specialized libraries of linked reads. Single-molecule approaches (optical maps (e.g., Bionano) and long-read sequencing, such as PacBio and Oxford Nanopore Technologies) allow for alignment of sequences from multiple individuals and, because of read length, enable characterization of sequences missing in the reference genome. Both of these approaches allow for the characterization of small- and intermediate-sized SVs. Large SVs (i.e., > 1 Mb) are more effectively characterized using optical maps (e.g., [83]). Collectively, these innovations have led to the most comprehensive characterization of CNV/PAV to date [84, 85]. However, the underlying data are still relatively expensive and must be generated at high depth for confident calls, making them impractical at the scale in which crop improvement programs often operate.

#### Characterizing structural variation through creation of a pan-genome reference

Access to multiple reference-quality genome assemblies within a species provides opportunities to identify SVs in a non-reference-biased manner. However, a number of challenges arise in such an approach. First, several crop species have large, complex genomes, making numerous assemblies per taxon cost-prohibitive. To overcome this limitation, a small number of breeding program founder individuals, which capture the majority of segregating haplotypes, can be targeted for genome assembly and identification of relevant SVs. Second, while multiple assemblies will reduce reference bias, assembly errors can lead to the detection of false SVs and compromise downstream analysis, particularly when de novo assemblies are generated using different data types or assembly algorithms. A third challenge is the consolidation of pan-genome variation into a single reference or coordinate system, a useful step for the analyses of the biological significance of SVs in crop species including QTL analysis, GWAS, and genomic prediction.

Several methods exist for summarizing SV information in a pan-genome context. One approach is to map resequencing reads to a reference genome, de novo assemble unmapped reads, and add the assembled contigs to the reference assembly (known as the map-to-pan approach) [48, 86]. This strategy can minimize errors by exploiting the information already available from a high-quality reference genome and limit the coordinate consolidation issue, but the genomic locations of newly assembled contigs remain unknown without further analysis. A second alternative is the construction of a graph-based rather than linear reference genome [87]. In this approach, any variant (SNP or SV) is added to the reference as a node at the genomic location where it is discovered [88, 89]. Recently, a hybrid approach between linear and graph-based reference genomes has been developed to build on the strengths of these methods. In this approach, reads are first mapped to a graph-based genome, and haplotypes are associated with one of the reference genomes used to build the graph. Reads are then realigned to this genome leading to more accurate mapping than the graph-based approach alone [90]. For detailed descriptions of each method, and their advantages and disadvantages, see [91, 92].

### Relevance of transposable elements to crop improvement

**Fig. 2:**
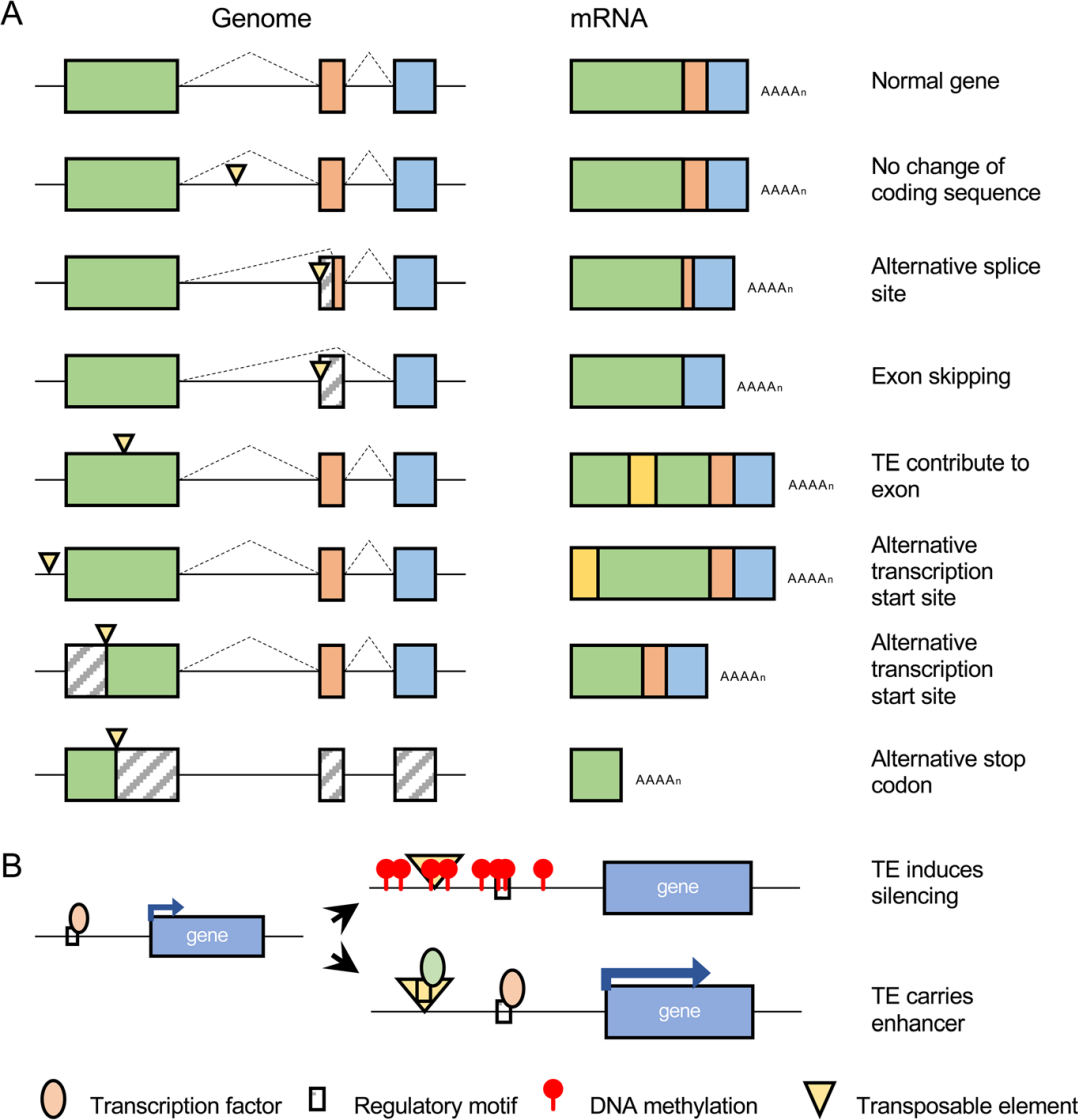
Functional consequences of new transposable element insertions. **a** Possible effects on gene product structure. **b** Possible effects on gene product abundance

As pan-genomes become widely available for crop species, TEs, a driver of structural variation, will receive increasing attention in crop improvement. Plant genomes (including crop species) are particularly rife with TEs [93], and the relevance of TEs to crop phenotypes has been repeatedly demonstrated. Transposable elements can be functionally relevant in a number of ways including modifying the structure and amount of gene product that is transcribed (Fig. 2 [14, 23, 35, 37, 94–100];). For example, in maize, a Harbinger-like DNA transposon represses the expression of the *ZmCCT9* gene to promote flowering under long-day conditions [37]. In rice, a Gypsy retrotransposon has been shown to enhance the expression of the *OsFRDL4* gene and promote aluminum tolerance [101]. Two Copia retrotransposons independently inserted into the promoter region of the orange *Ruby* gene, resulting in its enhanced expression and driving convergent evolution of the blood orange trait [102]. Finally, a Copia retrotransposon *Rider* has created polymorphism in the *SUN* locus resulting in the oval shape typical of the Roma tomato variety [103, 104]. Despite their prevalence and relevance to agronomic phenotypes, TEs have, until recently, been largely ignored in crop improvement efforts.

TEs create the majority of insertions and deletions in many crop genomes. For example, > 75% of large InDels (i.e., ≥ 100 bp) in both tomato [23] and soybean [24] pan-genomes consist of at least one TE. Across four maize lines, there is greater than 1.6 Gb of TE sequence that was found to segregate in just this narrow subset of genotypes [105]. Genome-wide variation in TE content at the species level has, until recently, been difficult to characterize because, as described above, the repetitive fraction of genomes has historically been poorly assembled, and there are challenges with accurate read alignment to these regions. Methods to characterize variation in TE content using short-read data [106] and whole-genome comparisons [105] are emerging and will help provide access to a new level of functional variation underlying agronomic phenotypes.

Once TE sequences are captured in de novo genome assemblies, a critical remaining challenge is an accurate annotation to the family level. Three general approaches are used. The first is homology-based using existing TE databases such as Repbase [107] and P-MITE [108]. This approach is quick because it uses annotations from other species, but is limited by the availability of such information and the extent to which TE sequences are conserved across species [109]. The second approach is based on the copy number of sequences [110–112] and is relatively fast and sensitive for the identification of high-copy number repeats. However, the specific annotation of a sequence is unknown (i.e., these could be large gene families, TEs, other types of repeats), and low-copy TEs are often missed. The limited classification information provided by this approach hampers biological inference and utility for crop improvement. The third approach is the de novo identification of TEs based on structural features. Structural annotation does not rely on existing TE libraries and is very sensitive. This method depends critically on knowledge of the diagnostic structural components of TEs and, when this knowledge is incomplete or imprecise, can result in inaccurate annotation [113]. Recently, efforts have been made to combine these approaches into a comprehensive solution for TE annotation. Such pipelines incorporate structural and homology information, repetitiveness, existing TE curations, and extensive filtering to generate high-quality de novo TE annotations. Methods developed based on this approach include EDTA [114] and RepeatModeler2 [112]. Comprehensive TE annotation of high-quality pan-genomes will allow us to further explore their varied roles within crop genomes [115] and to link TE variation, a pervasive form of SV, to phenotypes with agronomic relevance [116].

### Advancing QTL mapping and GWAS using crop pan-genomes

Two main approaches are used to identify genomic regions associated with a desired phenotype: QTL mapping with biparental populations and GWAS with panels of diverse individuals. Early crop reference genome assemblies facilitated the development of platforms (e.g., Illumina SNP chips) that allow for rapid, cost-effective genotyping of thousands or millions of SNPs across large sets of individuals. This increase in marker density dramatically increased resolution in mapping studies, which aided in the identification and cloning of QTLs associated with disease resistance, drought tolerance, yield, plant architecture, and other important agronomic traits [117]. With these marker-trait associations identified, breeders can use linked markers to select the best plants in a population without extensive phenotyping, either as functional markers [118] or through marker-assisted selection [119].

**Fig. 3:**
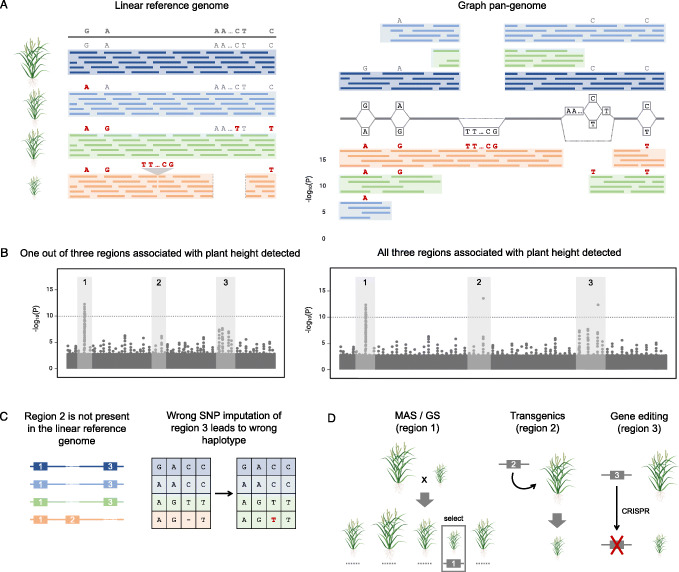
Impact of pan-genome representation on dissection of quantitative variation and applications to crop improvement. **a** Mapping reads to a single reference genome assembly (left) or a pan-genome graph (right) that captures structural variation in the species. **b** Impact of the read mapping method (single reference assembly vs. pan-genome graph) and subsequent variant calling on the ability to dissect the genetic architecture of a trait. **c** Causes for lack of identification of significant regions of the genome using variants called by mapping reads to a single reference genome assembly. **d** Methods breeders can utilize to exploit newly identified variants involve marker-assisted selection (MAS) and/or genomic selection (GS), inserting sequence through a transgene, or making other changes to a causative region with genome editing

One major concern in QTL mapping or GWAS based on a single reference genome is reference bias [120]. If variants associated with a trait are not present in the reference genome, then QTL mapping or GWAS will not be able to detect them (Fig. 3a, b). For example, a maize gene conferring resistance to sugarcane mosaic virus could be identified by GWAS using markers based on the B73, but not the PH207, genome assembly, because the gene was not present in the PH207 assembly [120]. This situation is further exacerbated with more diverse germplasm (i.e., secondary gene pools), making it difficult to identify causative variation and bring it into the germplasm of breeding programs. A further problem is that true deletions relative to a reference genome are indistinguishable from missing data due to technical problems (e.g., low sequence coverage). Imputation of allelic variants across true deletions can result in decreased power to detect a significant association (Fig. 3c).

QTL and GWAS studies have primarily relied on SNP data to date, but other markers have been useful in linking different types of variation to phenotype. For example, GWAS performed with both read depth variants (RDVs, a proxy for SVs), and SNPs in maize demonstrated that RDVs were enriched for significant GWAS results relative to SNPs for traits such as leaf development and disease resistance [77]. Similarly, in a large-scale GWAS using transcript abundance as a marker, gene associations with maize development traits were identified that were not detected by GWAS using SNPs [10]. While read depth and transcript abundance variants were useful in the initial efforts to assess the importance of SVs to phenotypic variation, they do not capture the complete structural variant landscape within a population. For example, read depth variants can only capture SVs that are present in the reference genome (e.g., insertions relative to the reference are not evaluated), leading to a strong reference bias and an incomplete picture of the relationship between SVs and phenotypes. RNA-seq is focused only on transcribed regions, is dependent on what tissues and developmental stages are sampled, and can be driven by both allelic variation in regulatory regions and true structural variation.

As the crop improvement paradigm shifts to a pan-genome perspective, the contribution of SVs to trait variation is becoming clear. Recently in *Brassica napus*, GWAS was performed with PAVs identified from eight whole-genome assemblies, and causal associations between SVs and silique length, seed weight, and flowering time were discovered that were not captured by SNP-GWAS. Likewise, GWAS based on the graph soybean pan-genome identified a PAV associated with variation in seed luster [24]. In peach, candidate causative SVs for early fruit maturity, flesh color around the stone, fruit shape, and flat shape formation have also been observed [121]. However, our understanding of the importance of SVs to phenotypic trait variation is still in its infancy. As technology and algorithm advancements allow for the complete SV landscape to be characterized at the scale of breeding programs and incorporated into a graph-based framework, it is anticipated that we will see a growing number of SVs underlying phenotypic variation important for crop improvement.

### Advancing genomic prediction using crop pan-genomes

A number of important traits for crop improvement are controlled by many QTLs with small effect (e.g., yield). A complex genetic architecture makes it difficult to identify all QTLs underlying a trait, correctly estimate their effects, and introgress them into elite lines using methods such as marker-assisted selection [122–124]. Genomic selection is an alternative approach for complex traits, where marker effects are estimated from a training set, the phenotype of an individual is predicted based on the estimated marker effects (i.e., genomic prediction), and selections are made based on the predicted phenotype [125]. Regression and Bayesian approaches for genomic prediction were first described in the early 2000s and revolutionized animal and plant breeding [126]. Using SNPs as predictors, important agronomic traits such as grain yield, grain moisture, grain quality, biomass traits, and stalk and root lodging have been predicted with fairly high accuracy [127–133].

Traditionally, SNPs identified relative to a single reference genome have been used for genomic selection. However, as described above, there are a number of limitations and biases that are introduced with the use of a single reference for such applications. New approaches for identifying markers within a pan-genome framework are needed to improve prediction accuracy. The Practical Haplotype Graph (PHG) is one such method that successfully deals with the complexity of a species’ pan-genome at the scale necessary for complex traits and plant breeding programs [134]. In the PHG approach, existing genomic resources of breeding program founder lines (e.g., whole-genome resequencing data and/or whole-genome assemblies) are loaded into a graph-pan-genome database. Accurate imputation of low-sequence-coverage individuals (as low as 0.01× coverage) in the breeding population is achieved based on consensus haplotypes derived from the graph-pan-genome database. The PHG is a promising strategy for reducing the costs of genotyping, while also capturing a greater breadth of diversity in large breeding populations.

A major issue in genomic prediction is that genotype by environment (G×E) interactions decrease the prediction accuracy for individuals grown in novel environments. Statistical models that account for G×E have been designed to attempt to overcome this limitation [135–137]. Incorporation of SV data in such prediction models may further help to address issues of G×E in genomic prediction accuracy, because these variants have been shown to play a particularly important role in adaptation across environments. Not all SVs will be tagged by SNPs [70, 77, 138] and phenotypic variation driven by untagged SVs will be missed by prediction models. For example, Lyra et al. found that predictive ability for maize plant height under low nitrogen increased when adding just a few hundred CNVs to an analysis of ~ 20k SNPs [139]. However, while adding these additional markers may result in higher predictive accuracy, their addition may not be practical in breeding programs at the moment, as they require novel data generation and analysis infrastructure. Breeders need to balance the costs of scoring different markers with the increased efficiency of genomic prediction and genetic gain. For the time being, structural variation information from a pan-genome will be most readily used by breeders if existing SNP genotyping technology includes markers in strong linkage disequilibrium (LD) with phenotypically important SVs. For SVs not tagged by SNPs [70, 77, 138], characterization of these variants using novel approaches is only prudent if the genetic gain is large enough to justify the increased cost.

### Future challenges and opportunities in applications of pan-genomics for crop improvement

Beyond the promise that recent genomic advances offer for characterizing diversity in model crop systems and for improvement of trait mapping and prediction, they also present opportunities to tackle difficult and understudied crop genomes and could potentially enable novel, gene-editing approaches to breeding.

#### Complexity of polyploid genomes

Allopolyploidy (the result of interspecific or intergeneric hybridization and chromosome doubling) and autopolyploidy (the result of whole-genome duplication) are particularly common in plant species [140, 141]. In fact, all angiosperms have undergone at least two rounds of polyploidy in their evolutionary history [142]. Many have returned to a diploid state, bearing remnants of this evolutionary history in their genomes [143]. As a natural mechanism, polyploidization can increase allelic diversity, expand the complement of genes, generate novel phenotypic variation, and aid in adaptation to new environments [144, 145]. Taking advantage of this, plant breeders have also generated artificial polyploids resulting in increased grain yield [146], fruit size [147], and seedless fruit [148].

**Fig. 4:**
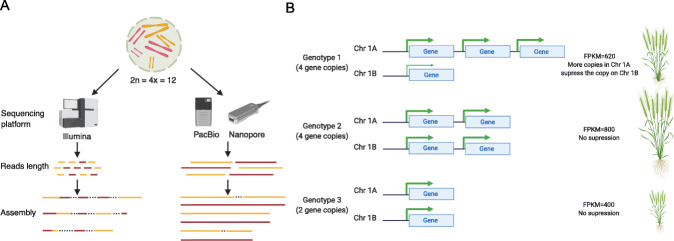
Impacts of technological advances to facilitate crop improvement in polyploid species. **a** Impact of sequencing technology on polyploid assembly. **b** Example of how understanding of a biological process is facilitated by having structural variation within subgenomes resolved beyond simply characterizing the number of copies in the genome

While polyploid crops are vitally important to sustain human life, genomic studies in these species have traditionally been very challenging for a number of reasons. High-quality genome assembly of polyploid species has been difficult to achieve due to their inclusion of multiple, closely related subgenomes and the associated challenges in discriminating homeologous loci and creating non-mosaic subgenome scaffolds (Fig. 4a). Many have resorted to sequencing diploid progenitors [149] or closely related species [58, 150] of polyploid crops in order to reduce genome complexity when generating initial reference assemblies. However, closely related diploids fail to capture lineage-specific SNPs, SVs, and other forms of variation that have accumulated post-polyploidization [39]. Beyond these difficulties in genome assembly, genomic approaches to polyploid crop improvement face further complications: (1) dissection of the genetic architecture of complex traits can be confounded when variants are not mapped to the correct subgenome [151, 152], a technical limitation, and (2) biologically, the more extensive epistatic interactions in polyploids [153, 154] and regulatory feedback between subgenomes can complicate the accurate prediction of phenotype based on genotype [155](Fig. 4b).

Advances in sequencing technologies and assembly algorithms are already addressing technical challenges in crop genomic research in polyploids [156]. Long-read sequencing with low error rates (e.g., PacBio HiFi reads) has made high-quality polyploid genome assembly possible, with recent assemblies containing fewer gaps and resolved homeologous scaffolds (Fig. 4a). Long-read assemblies now exist for polyploid crop species such as peanut [157], wheat [60], oilseed [22], and strawberry [158]. In some instances (e.g., potato [159]), multiple genome assemblies already exist within species. Nascent polyploid pan-genome studies are uncovering substantial diversity across species. For example, the de novo assembly of a single wheat cultivar captured 107,891 genes, and a map-to-pan assembly of 17 additional cultivars captured ~ 30,000 novel genes [53, 60]. As pan-genomic studies expand in polyploid crop species, we expect that, due to genomic redundancy and complexity, the degree of structural variation within polyploid species will be greater than that observed in diploid species, and SVs may be particularly fruitful markers for genomic approaches to polyploid crop improvement. Technical progress in assembling polyploid genomes (e.g., improvements to haplotype and homeolog phasing) should facilitate basic, biological study of the differences in the genotype-to-phenotype map between diploids and polyploids, knowledge of fundamental importance to the future of polyploid crop improvement.

#### Genomic resources for understudied crop species

For understudied crops, pan-genome-assisted breeding efforts remain limited due to the small size of the research communities for these species and, in some cases, due to the challenges associated with genome complexity. For the majority of understudied crop species, transcriptome assemblies are currently used as a proxy to the genome for improvement efforts. One such example is *Silphium integrifolium*, a species with a large genome size (2*n* = 2*x* = 14; haploid genome size of ~ 9 Gb [160];) that is currently being domesticated into an oil crop. Through transcriptome assembly and resequencing of 68 wild *S. integrifolium* accessions, several loci associated with adaptation to different climate conditions were identified [161]. While SNP data helped identify loci under selection, structural variation, an important source of local adaptation, remained uncharacterized. Pennycress (*Thlaspi arvense*) is another species that is currently being domesticated for use as an oil crop [162]. While it has advanced from an initial transcriptome assembly [163] to a full genome assembly [164], access to pan-genome variation is not yet available, despite the relatively small size (539 Mb) and simple genome structure of the species. Turfgrass and forage crops are further examples of understudied crops with limited genomic resources. Perennial ryegrass (*Lolium perenne*) has a fragmented draft genome [165], which may not be sufficient to enable pan-genomic research within the species. For other turfgrass species, such as hexaploid hard fescue (*Festuca brevipila*), long-read sequencing of the transcriptome has been used as a proxy of the reference genome, but it remains difficult to distinguish homeologs using this approach [166].

While pan-genomic studies may be in their infancy in non-model crops, it is anticipated that rapid advances in sequencing, assembly algorithms, and analysis pipelines in model systems and diminishing costs will very quickly enable this research. The time from publication of the first rice genome assembly to release of the first rice pan-genome was over a decade ([3]; Table 1). We anticipate that the development of genomic resources, including pan-genomes, will now be much more rapid. Indeed, pan-genomic studies have already been published in *Capsicum* (pepper) and *Juglans* (walnut) species [46, 51], and others will soon follow.

#### Rapid domestication of new and existing species

The recent availability of high-quality genomes and pan-genomes has enabled a new era of crop domestication. With pan-genome information, breeders can more effectively identify causal genetic variants (e.g., SNPs, CNV, PAV) underlying domestication traits and apply gene-editing tools to rapidly achieve desirable agronomic traits in wild plants. For example, the tomato pan-genome has revealed that variation at the fruit weight QTL *fw3.2* is caused by tandem duplication of the cytochrome P450 gene *SlKLUH* [23] rather than a SNP in the gene’s promoter as proposed earlier [167]. CRISPR/Cas9 gene editing to reduce the copy number of the *SKILUH* gene successfully altered fruit weight, a crop domestication phenotype [23]. Similarly, by using resequencing data and a map-to-pan approach, Gao et al. conducted a comparative analysis of 725 cultivated tomatoes and close wild relatives, uncovering gene loss during tomato domestication [49]. Further enrichment analysis suggested that defense response genes and nearly 1200 promoter sequences were targeted by selection during domestication and improvement [49]. A non-reference ~ 4 kb substitution in the *TomLoxC* promoter region was also discovered that modifies fruit flavor [49]. These variants that distinguish crops from their wild relatives are prime targets for gene editing for rapid domestication.

Domestication has greatly reduced the genetic diversity of crops compared to their wild relatives [168]. Identifying and utilizing genetic diversity from crop wild relatives has been a major focus in crop improvement [169, 170]. Together, pan-genome information and CRISPR/Cas9 technologies enable de novo domestication of wild plants and can reduce barriers to the use of genetic variation from secondary and tertiary gene pools (wild relatives) [171, 172]. For example, Zsögön et al. edited six loci in wild tomato (*Solanum pimpinellifolium*) and significantly increased its yield, productivity, and nutritional value resulting in de novo domestication of tomato [173].

In summary, the complete catalog of variation that has been made possible by recent genomic technology and a pan-genome approach presents a substantial opportunity for crop improvement. We can, not only move beyond single-reference-based resequencing in model crops to a full understanding of structural variation and its link to phenotype, but also tackle complex, polyploid genomes, rapidly move understudied crops into the genomic era, and bring down barriers between crops and their wild relatives so that breeders can more easily expand their tool kit to include exotic germplasm. While further infrastructure and method development is necessary to fully realize this potential, there is a paradigm shift in the making.

## Supplementary Information

**Additional file 1.** Review history.

## Data Availability

Not applicable
