# Supplementary material for: How the pan-genome is changing crop genomics and improvement
Source: Genome Biol. 2021 Jan 4;22:3. doi: 10.1186/s13059-020-02224-8 (PMC7780660; doi:10.1186/s13059-020-02224-8)
Supplement: Supplementary file 1 — Additional file 1. Review history. [file 13059_2020_2224_MOESM1_ESM.docx]

Review History

**First round of review**

**Reviewer 1**

**Comments to author:**

Della Coletta et al. present a review to describe recent progresses on structural variation and pan-genome characterization of crop plants and to discuss future challenges and opportunities in applying pan-genomics for crop improvement. This comprehensive review is nicely written. My comments are mainly minor.

Table 1 is largely duplicated with Table 1 of Bayer et al. published recently (https://www.nature.com/articles/s41477-020-0733-0/tables/1).

Line 44-45: The types of structural variants described here are not consistent with those shown in Figure 1, although SVs shown in Figure 1 can be categorized into the SV types described here. It's better to describe the SVs shown in Figure 1 clearly here. In addition, Figure 1 only shows tandem duplication. How about segmental duplication?

Line 197: Suggest to replace reference 91 with this one: https://science.sciencemag.org/content/319/5869/1527.abstract

Line 267-271: A recent study on peach GWAS using SVs should be added here: https://genomebiology.biomedcentral.com/articles/10.1186/s13059-020-02169-y

Line 284: change "bayesian" to "Bayesian".

Line 316: change "LD" to "linkage disequilibrium (LD)".

Line 333-334: seedless fruits (triploid watermelon and banana) are other good examples of artificial polyploids and should be included here.

**Reviewer 2**

**Comments to author:**

The authors present a really nice review on the recent advances in plant genomics, particularly on the usefulness and future directions of pan-genomes. The manuscript is well-written for a broad audience and I foresee this serving as a really useful resource for the community. I anticipate that it will be nicely cited.

I do offer two very minor suggested edits / comments.

1. Line 160-161 "First, crop species often have large, complex genomes, making numerous assemblies per taxon cost prohibitive"

Comment: I would suggest changing this to either "First, crop species may have large, ..." or "First, several crop species have large, ...". The maize genome is slightly larger than the average genome size for angiosperms. Yes, it's significantly larger than Arabidopsis and Rice, but is significantly smaller than a crop like Tulip (>30Gb) as well as tens of thousands of wild species. I don't think there is evidence that supports that crops tend to have larger genome sizes.


2. Line 328-330. "In fact, it is estimated that 80 percent of angiosperms are currently polyploids [128], and many others were once polyploids before returning to a diploid state, bearing remnants of this evolutionary history in their genomes [129]."

Comment: Not "many others were once polyploids", but rather all angiosperms have undergone at least two rounds of polyploidy in their evolutionary history. See https://www.nature.com/articles/nature09916?page=28  The subsequent line needs to be rewritten also.

**Authors Response**

**Point-by-point responses to the reviewers’ comments:**

Reviewer 1

*Della Coletta et al. present a review to describe recent progresses on structural variation and pan-genome characterization of crop plants and to discuss future challenges and opportunities in applying pan-genomics for crop improvement. This comprehensive review is nicely written. My comments are mainly minor.*

*Table 1 is largely duplicated with Table 1 of Bayer et al. published recently (*[*https://www.nature.com/articles/s41477-020-0733-0/tables/1*](https://www.nature.com/articles/s41477-020-0733-0/tables/1)*).*

**Author Response: We appreciate the concern that the reviewer brings up. We have tried to make our table different from those in previous reviews of this type (including the one that the reviewer highlighted) by including and ordering by the genome size and including information on the sequencing method. There is an ever expanding list of entries to be included in such a table as evidenced by the Apple paper that came out during the time this manuscript was under review. This is now included in our table and is not included in the table within the Nature Plants review. We also have a number of entries in our table that are from wild species (e.g. wild cabbage, Wild rice, and African rice) that are unique to our table.**

*Line 44-45: The types of structural variants described here are not consistent with those shown in Figure 1, although SVs shown in Figure 1 can be categorized into the SV types described here. It's better to describe the SVs shown in Figure 1 clearly here. In addition, Figure 1 only shows tandem duplication. How about segmental duplication?*

**Author Response: Thank you for pointing out this inconsistency. We have modified Figure 1 to now be grouped by presence/absence variation, copy number variation, and chromosomal rearrangements.**

*Line 197: Suggest to replace reference 91 with this one: https://science.sciencemag.org/content/319/5869/1527.abstract*

**Author Response: Thank you for this suggestion, we have made this substitution.**

*Line 267-271: A recent study on peach GWAS using SVs should be added here: https://genomebiology.biomedcentral.com/articles/10.1186/s13059-020-02169-y*

**Author Response: This is a very nice study that came out shortly after we submitted and definitely should be included. We have added a sentence describing these results as suggested.**

*Line 284: change "bayesian" to "Bayesian".*

**Author Response: This change has been made.**

*Line 316: change "LD" to "linkage disequilibrium (LD)".*

**Author Response: This change has been made.**

*Line 333-334: seedless fruits (triploid watermelon and banana) are other good examples of artificial polyploids and should be included here.*

**Author Response: Thank you for this suggestion. We have added this to the sentence.**

Reviewer 2

*The authors present a really nice review on the recent advances in plant genomics, particularly on the usefulness and future directions of pan-genomes. The manuscript is well-written for a broad audience and I foresee this serving as a really useful resource for the community. I anticipate that it will be nicely cited.*

*I do offer two very minor suggested edits / comments.*

*1. Line 160-161 "First, crop species often have large, complex genomes, making numerous assemblies per taxon cost prohibitive"*

*Comment: I would suggest changing this to either "First, crop species may have large, ..." or "First, several crop species have large, ...". The maize genome is slightly larger than the average genome size for angiosperms. Yes, it's significantly larger than Arabidopsis and Rice, but is significantly smaller than a crop like Tulip (>30Gb) as well as tens of thousands of wild species. I don't think there is evidence that supports that crops tend to have larger genome sizes.*

**Author Response: This is a very valid point that the reviewer brings up. We have changed this sentence accordingly.**

*2. Line 328-330. "In fact, it is estimated that 80 percent of angiosperms are currently polyploids [128], and many others were once polyploids before returning to a diploid state, bearing remnants of this evolutionary history in their genomes [129]."*

*Comment: Not "many others were once polyploids", but rather all angiosperms have undergone at least two rounds of polyploidy in their evolutionary history. See https://www.nature.com/articles/nature09916?page=28 The subsequent line needs to be rewritten also.*

**Author Response: Thank you for pointing this out and providing this alternative citation. We have modified this section to reflect this evolutionary history of angiosperms more accurately.**
